# Potential impact of reduced tobacco use on life and health expectancies in Belgium

**DOI:** 10.1007/s00038-019-01315-z

**Published:** 2019-11-28

**Authors:** Martina Otavova, Herman Van Oyen, Renata T. C. Yokota, Rana Charafeddine, Luk Joossens, Geert Molenberghs, Wilma J. Nusselder, Hendriek C. Boshuizen, Brecht Devleesschauwer

**Affiliations:** 1 Unit of Epidemiology, Biostatistics and Biodemography, Department of Public Health, University of Southern Denmark, 5000 Odense, Denmark; 2 Interdisciplinary Center on Population Dynamics, Department of Public Health, University of Southern Denmark, 5000 Odense, Denmark; 3 Department of Epidemiology and public health, Sciensano, Rue J Wytsman 14, 1050 Brussels, Belgium; 4 Department of Public Health and Primary Care, Faculty of Medicine, Ghent University, Ghent, Belgium; 5 Association of European Cancer Leagues, Brussels, Belgium; 6 I-BioStat, Universiteit Hasselt & KU Leuven, Hasselt, Belgium; 7 Department of Public Health, Erasmus MC, University Medical Center Rotterdam, Rotterdam, The Netherlands; 8 Department of Statistics, Informatics and Mathematical Modeling, Centre for Nutrition, Prevention and Health Services, National Institute for Public Health and the Environment, Bilthoven, The Netherlands; 9 Department of Veterinary Public Health and Food Safety, Faculty of Veterinary Medicine, Ghent University, Merelbeke, Belgium

**Keywords:** Smoking, Healthy life years, Unhealthy life years, DYNAMO-HIA, Smoking intervention

## Abstract

**Objectives:**

We investigated the potential impact of reduced tobacco use scenarios on total life expectancy and health expectancies, i.e., healthy life years and unhealthy life years.

**Methods:**

Data from the Belgian Health Interview Survey 2013 were used to estimate smoking and disability prevalence. Disability was based on the Global Activity Limitation Indicator. We used DYNAMO-HIA to quantify the impacts of risk factor changes and to compare the “business-as-usual” with alternative scenarios.

**Results:**

The “business-as-usual” scenario estimated that in 2028 the 15-year-old men/women would live additional 50/52 years without disability and 14/17 years with disability. The “smoking-free population” scenario added 3.4/2.8 healthy life years and reduced unhealthy life years by 0.79/1.9. Scenarios combining the prevention of smoking initiation with smoking cessation programs are the most effective, yielding the largest increase in healthy life years (1.9/1.7) and the largest decrease in unhealthy life years (− 0.80/− 1.47).

**Conclusions:**

Health impact assessment tools provide different scenarios for evidence-informed public health actions. New anti-smoking strategies or stricter enforcement of existing policies potentially gain more healthy life years and reduce unhealthy life years in Belgium.

**Electronic supplementary material:**

The online version of this article (10.1007/s00038-019-01315-z) contains supplementary material, which is available to authorized users.

## Introduction

Smoking is the leading risk factor for preventable and premature mortality (WHO 2017). Smoking is linked to ill-health and disability as it contributes to the pathogenesis of several chronic diseases, such as cancer, cardiovascular diseases and chronic respiratory diseases, and worsens already existing medical conditions (Ostbye et al. 2002; Strandberg et al. 2008). The harmful impact of smoking is mainly seen in late adulthood, but children, adolescents and young adults may also be affected in terms of quality of life or its length, i.e., tobacco use and secondhand smoke exposure reduce the disability-free life expectancy (Reuser et al. 2009; Aguilar-Palacio et al. 2018).

Health expectancies are summary measures of population health combining morbidity and mortality into a single indicator. Healthy life years, also known as disability-free life expectancy, is an indicator based on limitations in daily activities and measures how many years an individual at a particular age is, on average, expected to live without disability (Robine et al. 1999). Due to population aging, increasing healthy life years is a main policy objective in the EU and in several member states including Belgium (Lagiewka 2012; Obyn et al. 2017; Bogaert et al. 2018). There is, however, little evidence on how reduced tobacco use may contribute to achieving the EU policy goal of increasing healthy life years, as most studies focused on effects on disease-specific incidence and mortality, overall life expectancy, or health-adjusted life years (e.g., Holm et al. 2014; Lhachimi et al. 2016; Singh et al. 2019).

In 2005, Belgium ratified the WHO Framework Convention on Tobacco Control (WHO FCTC) (WHO 2016). Since then, many anti-smoking policies were implemented, and a slight but continuous reduction in the smoking prevalence has been observed (2004: 28%; 2008: 25%; 2013: 23%) (BHIS 2018). Belgian government banned smoking in the public and work places, restaurants, bars and schools. In 2017, the Walloon government approved a ban on smoking in cars in the presence of minors (children until 16 years of age). Smoking policies enforced by the WHO FCTC also include bans on tobacco advertising, promotion, and sponsorship, and changes in tobacco taxation policy. Furthermore, individual and partially reimbursed smoking cessation support is available at both primary care, hospitals, and health clinics. There is a toll-free quitting hotline available for discussing smoking cessation issues (WHO FCTC 2016).

Despite the recent progress in the development of smoking prevention and cessation programs in Belgium, the various anti-smoking laws and interventions lack a global vision, resulting in a drop from 13th (2013) to 17th place out of 35 European countries on the Tobacco Control Scale 2016 (TCS) in 2016 (Joossens and Raw 2017). There is therefore an urgent need for pro-active policy support to enable the development of a comprehensive anti-tobacco plan in Belgium.

In this study, we investigated the potential impact of various reduced tobacco use scenarios on life expectancy and health expectancies. The tobacco control scenarios were introduced as “what-if” scenarios (e.g., “what if every smoker quits smoking?”) or as policy/intervention scenarios (e.g., “what if a new nationwide policy is implemented raising the legal age to buy tobacco products from 16 to 18”)?

We used DYNAMO-HIA 2.0.7 to quantitatively compare the alternative scenarios with the “business-as-usual” scenario. To measure the impact on overall life expectancy (LE) and healthy (HLY) and unhealthy (ULY) life years, the software quantifies the effects of risk factor changes that are initiated by newly enforced interventions and policies. We used the tool to calculate the reduction in HLY due to smoking and the potential gains in HLY due to implementing new policies.

## Methods

### DYNAMO-HIA software

DYNAMO-HIA is a partial micro-simulation modeling tool combining a stochastic micro-stimulation to generate risk factor histories with a deterministic method for the disease life table to calculate disease, disability or survival probability. The tool applies an epidemiological model to estimate the net transition probabilities from risk factor prevalence, relative risk (RR) for death, and baseline all-cause mortality, assuming that the age-specific risk factor exposure does not change over time (Boshuizen 2010; Lhachimi et al. 2008, 2010). While several other tools exist for health impact assessment, as, for example, reviewed by Fehr et al. (2016), DYNAMO-HIA offers the advantage of being generic, flexible, and publicly available (via http://www.dynamo-hia.eu/).

DYNAMO-HIA software estimates the health impact of different policy scenarios over time, by comparing an alternative scenario with the “business-as-usual” scenario (Boshuizen et al. 2012; Lhachimi et al. 2012). Policies are modeled as changes in the risk factor prevalence or as changes in transition probabilities between the risk factor states (Lhachimi et al. 2012).

An updated version, DYNAMO-HIA 2.0, used for the simulation presented in this paper, models the impact of the risk factor prevalence on disability-free life expectancy directly by using the overall odds ratio (OR) of disability. In this case, a hazard ratio of (other-cause) disability is calculated by combining disability prevalence and overall odds ratio (OR) of disability. This modeling approach requires the following age- and gender-specific input data: (1) Belgian data on population structure, mortality rates and projection of newborns; (2) Belgian data on disability; (3) smoking prevalence in Belgium; (4) OR of disability quantifying the association between smoking and disability and RR of death quantifying the RR of smoking on total mortality.

Information on smoking prevalence and disability, based on the Global Activity Limitation Indicator (GALI), was obtained from the Belgian Health Interview Survey 2013.

### Belgian Health Interview Survey

The Belgian Health Interview Survey (BHIS) is a cross-sectional household interview survey, conducted periodically every 4 to 5 years, that aims to collect information on the health status of the Belgian population. Each survey includes approximately 10,000 individuals. The detailed methodology of the survey is described elsewhere (Demarest et al. 2013; Scientific Institute of Public Health 2013).

In 2013, 8850 households were contacted, of which 5049 participated (57%), yielding a sample of 10,829 individuals (Van der Heyden and Charafeddine 2013). Since the current analysis was restricted to individuals aged 15 years and older and excluded subjects with missing information on disability and smoking, our final sample included 6085 individuals.

### Global Activity Limitation Indicator

GALI is the underlying measure of the European indicator healthy life years (Robine et al. 2013). GALI is a single-item survey instrument that aims to identify individuals in a population who consider themselves as having long-term, health-related participation restrictions or limitations in their daily activities (Berger et al. 2014; Van Oyen et al. 2018). Three severity levels are considered: none, limited but not severely, and severely limited. Individuals are considered to be disabled when they report themselves as severely limited or limited but not severely, as is commonly done when computing the HLY indicator (Jagger et al. 2008; Jagger et al. 2010).

Total life expectancy (LE), healthy life years (HLY) and unhealthy life years (ULY) are the summary measures of population health calculated in our study. In DYNAMO-HIA software, LE, HLY and ULY are calculated either as cohort or cross-sectional life expectancies. Cohort life expectancies are calculated for a simulated cohort and based on a cohort life table that calculates the LE using cohort-specific birth and mortality rates stratified by age from birth (Imai and Soneji 2007). In DYNAMO-HIA, the number of years lived in a certain health state during the follow-up period by all the simulated individuals is summed up and divided by the total number of simulated people at baseline. Cross-sectional life expectancies are calculated based on Sullivan’s method. Sullivan’s life expectancy is a measure utilizing the mortality—the difference between the survival in the current year and the next year—from a period life table. To calculate this type of life expectancies for a particular year, DYNAMO-HIA uses a two-step process. First, it calculates mortality in that given year by taking the difference between the survival in the current year and the next year. Then, it combines mortality with disability prevalence in that year into a period life table that is based on information from the one calendar year. Cohort and Sullivan’s life expectancies are calculated for individuals on one-year category up to age 95 and a 95 + category (Boshuizen 2010).

### Smoking prevalence

Age- and gender-specific smoking prevalence for individuals aged 15 and above was derived from the BHIS 2013 in a multi-step process. First, multiple fractional polynomial models were fitted to accommodate the nonlinear association between age (continuous) and smoking status (current, former, never). Next, logistic regression was used to model smoking prevalence as a function of sex and the polynomial age terms, taking into account the complex survey design (Ambler 2015; Lumley 2018). Finally, the prevalence of each smoking status was internally normalized, so that three age- and sex-specific prevalence estimates summed to 100%. Smoking prevalence by age and sex is shown in Figure S1 (Online Supplement).

### Disability prevalence and odds ratios for disability

Age- and sex-specific disability prevalence and odds ratio of disability by smoking status based on the GALI were derived from the BHIS 2013 using logistic regression, corrected for age (continuous) and sex and taking the complex survey design into account. Models were fitted separately for men and women, and correction for age (continuous) and smoking status was included. The disability prevalence and the odds ratios are shown in Figure S2 and Table S1 (Online Supplement).

### Transition probabilities

Transition probabilities of smoking, i.e., starting rates, quit rates and restarting rates, were directly derived by DYNAMO-HIA from the input data—i.e., smoking prevalence, RR for all-cause mortality and baseline all-cause mortality (Boshuizen et al. 2012). These net transition rates were estimated such that the age-specific prevalence of smoking remained stable over time, i.e., in the future, the age distribution of smoking is assumed to be the same as the current smoking distribution by age (Boshuizen 2010; Van de Kassteele et al. 2012).

### Relative risks of mortality

Gender-specific relative risks (RRs) of smoking on total mortality for Belgium were obtained from a study by Charafeddine et al. (2012). The RRs are reported in Table S2 (Online Supplement).

### Demographic information

Population size and mortality rates by age and sex for the year 2016 were derived from Statistics Belgium (2018). The projection of newborns between 2018 and 2050 was derived from the United Nations World Population Prospects (2017).

### Simulated population

Our simulated population was based on the demographic characteristics of real-Belgian population listed above. We simulated 2500 individuals for each age class (0–95) and gender. In total, our simulated population size was 480,000 individuals.

### Scenarios

Potential impact of reduced tobacco use was modeled by introducing business-as-usual and multiple “what-if” and policy/intervention scenarios—characterized by either a change in (re)start or quit transition probabilities or in smoking prevalence. All scenarios were simulated and compared to the reference of “business-as-usual” scenario in terms of changes in healthy life years (HLY), unhealthy life years (ULY), and life expectancy (LE) and their projected prevalence of never, current and former smokers. The reference scenario was based on the current prevalence of never, current and former smokers, stratified by age and sex, and on the current transition rates between the risk factor groups in Belgium. The reference took into account only the currently existing smoking control policies, without any additional interventions. All scenarios are described in Table 1.

**Table 1:** Overview of reduced tobacco use scenarios and their comparison to the reference scenario

Scenario	Definition	
*Reference*		
“Business-as-usual” scenario	Current prevalence of never, current and former smokers, current transition rates between the risk factor groups and current existing smoking control policies in Belgium	

	Change compared to the reference scenario	Gains compared to the reference scenario
*“What*-*if” scenarios*		
1. Smoking-free population	Population consists of never smokers only	Quantification of full burden of smoking on the overall population health
2. Zero (re)start probabilities	(Re)start chances equal 0%	Maximum gains from smoking initiation interventions
3. All smokers quit	Quit chances equal 100%	Maximum gains from smoking cessation interventions
4. Zero (re)start probabilities and all smokers quit	(Re)start chances equal 0% Quit chances equal 100%	Maximum gains from smoking initiation and smoking cessation interventions combined
5. Smoking prevalence in Sweden	Prevalence of never, current and former smokers in Sweden in 2016	Quantification of the health burden of smoking if the smoking prevalence in Belgium matched the one of Sweden, a country with the lowest smoking prevalence in Europe according to OECD (OECD 2016)
*Policy/intervention scenarios*		
6. No smoking initiation before age 18	Zero smoking prevalence below age 18	Maximum gains from raising the minimum age for purchase of tobacco from 16 to 18 years
7. 30% increase in quit probabilities	Quit chances are multiplied by 1.3	The lower bound of an increase in quit probabilities if the smoking quit interventions are provided by medical personnel (Grignon and Reddock 2012)
8. Doubling quit probabilities	Quit chances are multiplied by 2.0	Quantification of the effect size of smoking cessation interventions as found in literature review (Lemmens et al. 2008; Levy et al. 2010; Minary et al. 2013)

## Results

### Effect of scenarios on smoking prevalence

**Fig. 1:**
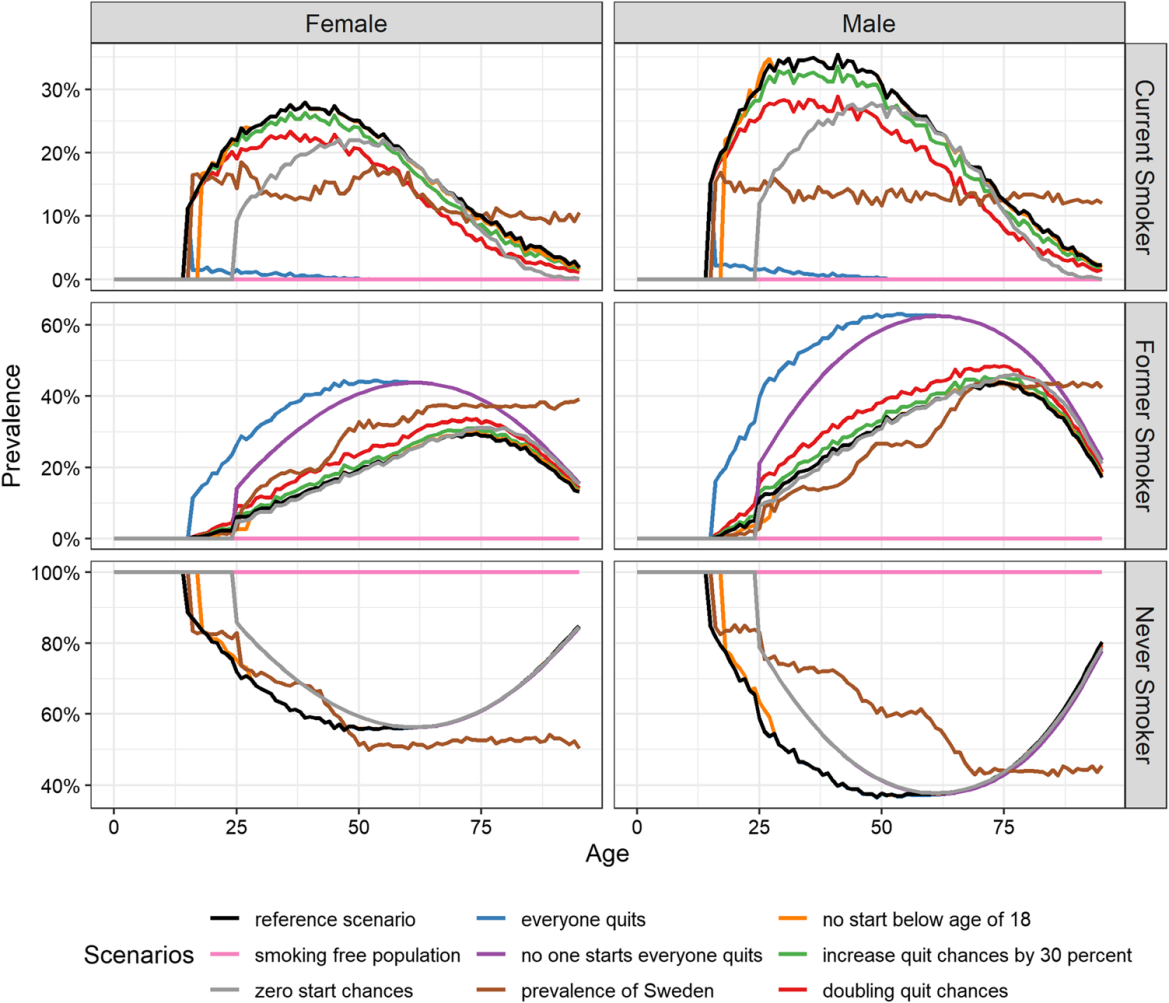
Smoking prevalence by age and gender. Effect of different reduced tobacco use scenarios in Belgium, 2028

Short- and long-term effects of alternative scenarios on the changes in prevalence of never, current and former smokers by age separately for the male and female populations for 2028 are given in Fig. 1 and for 2048 in Figure S4 (Online Supplement).

In the “business-as-usual scenario,” the prevalence of current smokers reflects the effect of smoking policies and interventions already in place. In the “smoking-free-population” scenario, the prevalence of never smokers is by definition 100% during the entire simulation period. The “zero (re)start probabilities” scenario, mimicking the maximum effect of smoking prevention programs, causes a reduction in the prevalence of current smokers as compared to the reference scenario. By 2028, this reduction is mainly observed among the younger ages and is mirrored by an increase in never smokers also at younger ages. Increased projection time allows the effect of this scenario to become observable among the middle and higher ages as the adolescents grow older and reach middle and late adulthood. The “all smokers quit” scenario, mimicking the maximum effect of smoking cessation programs, causes an immediate reduction in the prevalence of current smokers, mirrored by an increase in the prevalence of former smokers when compared to the “business-as-usual scenario.” This reduction in prevalence of current smokers is greater in the first years after the intervention and becomes stable after 30 years. By definition, this scenario does not affect the prevalence of never smokers. The same trend is observed in the short and long run. The “no smoking initiation before age 18” scenario reduces the prevalence of smokers among adolescents slightly, keeping this trend constant over time. The “30% increase in quit probabilities” scenario causes initially a small reduction in the prevalence of smokers at all ages but with time, larger reduction is observed in late adulthood. Similar pattern but more pronounced is observed for the “doubling quit probabilities” scenario.

### Effect of scenarios on healthy life years, unhealthy life years and life expectancy

We investigated the impact of the scenarios on the cross-sectional life expectancies for men and women at the age of 15 in 2028 and 2048 (Table 2).

**Table 2:** Impact of reduced tobacco use scenarios on healthy life years (HLY), unhealthy life years (ULY) and overall life expectancy (LE) (in years) for men and women at the age of 15 in 2028 and 2048

	HLY		ULY		LE	
	2028	2048	2028	2048	2028	2048
*Men*						
Reference scenario	50.07	49.97	14.04	14.01	64.11	63.98
*Difference with reference scenario*						
1. Smoking-free population	3.44	3.54	− 0.79	− 0.76	2.65	2.78
2. Zero (re)start probabilities	0.53	1.59	− 0.26	− 0.75	0.27	0.84
3. All smokers quit	1.73	1.74	− 0.72	− 0.74	1.01	1.00
4. Zero (re)start probabilities and all smokers quit	1.88	2.25	− 0.80	− 0.93	1.08	1.32
5. Smoking prevalence of Sweden	0.92	1.03	− 0.57	− 0.56	0.35	0.47
6. No smoking initiation before age 18	0.03	0.03	− 0.02	− 0.01	0.01	0.02
7. 30% increase in quit probabilities	0.14	0.26	− 0.04	− 0.09	0.10	0.17
8. Doubling quit probabilities	0.40	0.68	− 0.14	− 0.25	0.26	0.43
*Women*						
Reference scenario	51.65	51.51	17.32	17.34	68.97	68.85
*Difference with reference scenario*						
1. Smoking-free population	2.74	2.88	− 1.88	− 1.90	0.86	0.98
2. Zero (re)start probabilities	0.50	1.44	− 0.43	− 1.23	0.07	0.21
3. All smokers quit	1.55	1.58	− 1.37	− 1.40	0.18	0.18
4. Zero (re)start probabilities and all smokers quit	1.67	1.98	− 1.47	− 1.68	0.20	0.30
5. Smoking prevalence of Sweden	0.17	0.32	− 0.40	− 0.45	− 0.23	− 0.13
6. No smoking initiation before age 18	0.05	0.06	− 0.04	− 0.05	0.01	0.01
7. 30% increase in quit probabilities	0.12	0.21	− 0.09	− 0.18	0.03	0.03
8. Doubling quit probabilities	0.34	0.57	− 0.29	− 0.49	0.05	0.08

The “business-as-usual” scenario shows that in 2028 and 2048, 15-year-old men/women are expected to live additional 50/52 years without disability and 14/17 years with disability.

In comparison with the reference scenario, in 2028 for the male/female population, the “smoking-free population” scenario would result in an increase in HLY by 3.4/2.7 years, a decrease in ULY by 0.79/1.9 years and an increase in total LE by 2.7/0.86 years. By 2048, these differences become even more pronounced—HLY would increase by 3.5/2.9 years, ULY would decrease by 0.76/1.9 years and LE would increase by 2.8/0.98 years in men/women. For a cohort of 15-year-olds in 2018, the same scenario of absence of current and former smokers also results in maximum gains in terms of HLY and total LE: about 3.6 years in HLY and 2.8 years in overall LE in males and 2.8 years in HLY and 0.98 years in overall LE in women (Online Supplement). If Belgium had smoking prevalence of Sweden, the European country with the lowest smoking prevalence, the gain in terms of HLY and the reduction in ULY would be substantial for both genders in both reported years. For the 15-year-old male/female in 2028, HLY would increase by 0.92 and 0.17 years, ULY would decrease by 0.57 and 0.40 years. By 2048, the increase in HLY would be greater for both sexes, while an additional decrease in ULY would be observed for females only.

The highest gains among all “what-if” scenarios were reported for the “zero (re)start probabilities and all smokers quit” scenarios. In 2028, men would gain 1.9/1.1 years in HLY and LE and lose 0.80 years in ULY; women would gain 1.7/0.20 years in HLY and LE and lose 1.5 years in ULY. By 2048, this alternative scenario would gain 2.3/2.0 year in HLY for men/women and lose 0.93/1.7 years in ULY in men/women, respectively.

By increasing the quit probabilities by 30%, HLY would increase by 0.1/0.26 years for men by 2028 and 2048. For women, these differences would be 0.12/0.21 years by 2028/2048, respectively. Doubling the quit probabilities yields an increase of 0.40/0.34 years in HLY for men/women by 2028; the gains in HLY further increase up to 0.68/0.57 years for men/women by 2048. In both scenarios and for both sexes, ULY decreases gradually over time with an increase in quit probabilities. Although the gain in HLY is greater among males, the reduction in ULY is more notable among females. If the legal age of smoking rose from 16 up to 18, HLY would increase by 0.03 and 0.05 years, and ULY would decrease by 0.02 and 0.04 in 2028 for men/women. In 2048, only a slight increase in HLY and a decrease in ULY for women and almost no change for men would be observed.

The impact of “what-if” scenarios and smoking policy/interventions on the cohort life expectancies led to similar conclusions as for the impact on cross-sectional life expectancies (Table S3 Online Supplement).

## Discussion

To our knowledge, this is the first Belgian study aiming to compare how various reduced tobacco use scenarios may affect the length of HLY, ULY and overall LE of the Belgian population as a result of scenarios linked changes in the prevalence of current, former and never smokers. The comparison was made using DYNAMO-HIA, a dynamic population model for simulating the projection of real-life Belgian population for a period of 30 years to the future, separately for men and women (Lhachimi et al. 2012).

Our findings confirm results of prior studies that smoking is one of the main risk factors for disability and premature mortality in the male and female population both in international research and in publications with focus on Belgium (Brønnum-Hansen and Juel 2001; Ferrucci et al. 1999; Reuser et al. 2009; Van Oyen et al. 2014; Yokota et al. 2018). As most of our scenarios model maximum health gains, we provide valuable guidance to policy makers on which measures potentially have the highest impact. Furthermore, our study provides novel information on how reduced tobacco use can contribute to achieving the EU policy goal of increasing the number of HLYs (Lagiewka 2012).

Comparisons of modeled reduced tobacco use scenarios with the reference scenario indicate that the gains in HLY or LE and the reduction in ULY or in smoking prevalence differ in each scenario and over the projection period. The “smoking-free population” scenario demonstrates the greatest increase in HLY and LE and the greatest reduction in ULY for both genders among all scenarios; this scenario is, however, not realistically achievable, but rather reflects the current population level impact of tobacco use. The impact under the “zero (re)start probabilities” scenario is built up over time and is more effective in the future than in the short run. Interventions preventing smoking initiation mainly focus on never smokers among adolescents who possess low absolute risks of disability and mortality, and hence, their gains in terms of health are more observable in further future as the adolescents reach later adulthood.

The smoking control policies focusing on smoking cessation, maximized in the “all smokers quit” scenario, result in larger gains in HLY and LE in the first years after the interventions are implemented and their effect size remains almost constant over the next 30 years. These results confirm the findings by Kulik et al. (2012) that intervention/policies targeting smoking cessation are more effective in the short and long term than programs focusing on the prevention of smoking initiation.

Our results showed that policy/intervention methods combining prevention of smoking initiation with smoking cessation programs are the most effective among all the alternative scenarios, yielding the largest decrease in the smoking exposure and in ULY as well as the largest increase in HLY and total LE. The reduction in ULY is even greater for the “zero (re)start probabilities and all smokers quit” scenarios than for the “smoking-free population” scenario between the years 2018 and 2048 in the male population. A possible explanation is that individuals in the smoking-free population without any smoking histories accumulate more ULYs over their prolonged overall life course than individuals in a population consisting of never and former smokers. The fact that a combination of two different potential strategies for eradication of smoking is the most effective one is supported by findings from Rose that policies targeting the whole population are often the most effective ones (2001).

Our results indicate that an implementation of a nationwide policy raising the legal age limits to buy tobacco products from 16 to 18 would only result in a negligible reduction in smoking prevalence among young people and in turn to an increase in HLY and LE. These claims support findings of Fidler and West who investigated the impact on smoking prevalence after raising the minimum age of legal access to tobacco products from 16 to 18 in 2007 in England (2010). As adolescence is a sensitive developmental period, many risk factor behaviors peak during this time (Office of the Surgeon General 2012). Preventing young people from experimenting with tobacco products when they are the most vulnerable should become priority of the policy makers in the government.

### Strengths and limitations

An important strength of our study is the use of nationally representative data from the Belgian population. Added value to this study also includes the use of disability indicator based on the GALI, allowing better comparability with international studies that use the same instrument. The key strength of our study relates to the use of a dynamic modeling tool exclusively developed for health impact assessment. DYNAMO-HIA software can distinguish different risk factor states in order to generate transition probabilities between these states necessary for modeling the impacts of various interventions/policies on population health.

Our study has several limitations that must be considered when interpreting the results. Self-reported data on disability and smoking behavior were obtained from cross-sectional survey, and thus, assessing the causal relationship between smoking and disability prevalence may result in a temporal bias. Also, selection bias and underestimating of the true smoking exposure may have occurred in the BHIS 2013 and be further aggravated by the exclusion of individuals with missing information on smoking or the GALI.

The BHIS 2013 did not provide information on time since quitting for the former smokers; hence, the OR quantifying the association between smoking and disability does not take into account such information. Prior studies report conflicting findings on the impact of smoking cessation on disability. Some suggest that former smokers have similar disability hazards as current smokers, while others suggest that the smoking duration and time since quitting significantly affect the health-related quality of life and need to be considered (Ostbye et al. 2002; Reuser et al. 2009; Sarna et al. 2008).

We calculated health expectancies by Sullivan’s method. This approach assumes constant transition rates between disability states and in case of rapid and sudden changes in the observed period may lead to biased results (Sullivan 1971). Prior studies showed that the Sullivan method cannot detect sudden changes in disability transition rates, but can still provide good estimates if the changes in disability prevalence are smooth and relatively regular over longer period of time (Mathers 1997). When comparing the results with the cohort life table approach, however, conclusions appeared to the robust.

The main drawback of our study is the lack of uncertainty quantification provided by DYNAMO-HIA. In its current form, the software does not include probabilistic sensitivity analysis as its implementation into the model would be time-consuming and cost intensive.

### Conclusions

Our findings provide a better understanding of how a reduction in tobacco use may affect HLY, ULY and LE. We showed that the nationwide anti-smoking policies/interventions, combining the prevention of smoking initiation with the smoking cessation programs, are the most beneficial in reducing smoking prevalence and in turn increasing HLY and decreasing ULY in both the short and long runs. Future research should explore the role of frequency of smoking and time since quitting in the impact of tobacco control interventions on health expectancies. Nonetheless, we can conclude that all modeled scenarios reduce the prevalence of smoking and prolong the years without disability.

## Electronic supplementary material

Below is the link to the electronic supplementary material.

Supplementary material 1 (PDF 733 kb)
