# Supplementary material for: Potential impact of reduced tobacco use on life and health expectancies in Belgium
Source: Int J Public Health. 2019 Nov 28;65(2):129–38. doi: 10.1007/s00038-019-01315-z (PMC7049546; doi:10.1007/s00038-019-01315-z)
Supplement: Supplementary file 1 — Supplementary material 1 (PDF 733 kb) [file 38_2019_1315_MOESM1_ESM.pdf]

International Journal of Public Health – Supplementary Material

Potential impact of reduced tobacco use on life and health expectancies in Belgium

Supplementary Tables

Table S1. Odds ratios (OR) quantifying the association between smoking and disability, based on the Belgian Health Interview Survey 2013.

	OR current smoker	OR former smoker
Men	1.78	1.14
Women	2.31	1.21

Table S2. Relative risks (RR) quantifying the association between smoking and mortality[1].

	RR current smoker	RR former smoker
Men	2.13	1.35
Women	1.42	1.23

[1)] Charafeddine R, Van Oyen H, Demarest S (2012) Does the association between smoking and mortality differ by educational level? Social Science & Medicine 74(9):1402-1406.

Table S3. Impact of "what-if" scenarios and smoking policy/interventions on healthy life years, overall life expectancy, and unhealthy life years (in years) of a cohort of 15-year-olds in Belgium in 2018.

	Men			Women		
	HLY	*ULY*	*LE*	*HLY*	*ULY*	*LE*
**Reference scenario**	49.89	14.02	63.91	51.59	17.23	68.92
Difference with reference scenario						
1. Smoking-free population	3.62	-0.78	2.84	2.79	-1.81	0.98
2. Zero (re)start probabilities	2.60	-0.56	1.84	1.96	-1.27	0.69
3. All smokers quit	1.76	-0.75	1.01	1.48	-1.30	0.18
4. Zero (re)start probabilities and all smokers quit	2.98	-0.76	2.22	2.35	-1.63	0.72
5. Smoking prevalence of Sweden	1.11	-0.57	0.54	0.29	-0.37	-0.08
6. No smoking initiation before age 18	0.03	-0.02	0.05	0.08	-0.03	0.05
7. 30% increase in quit probabilities	0.26	-0.09	0.17	0.22	-0.18	0.04
8. Doubling quit probabilities	0.74	-0.27	0.47	0.55	-0.46	0.09

Supplementary Figures

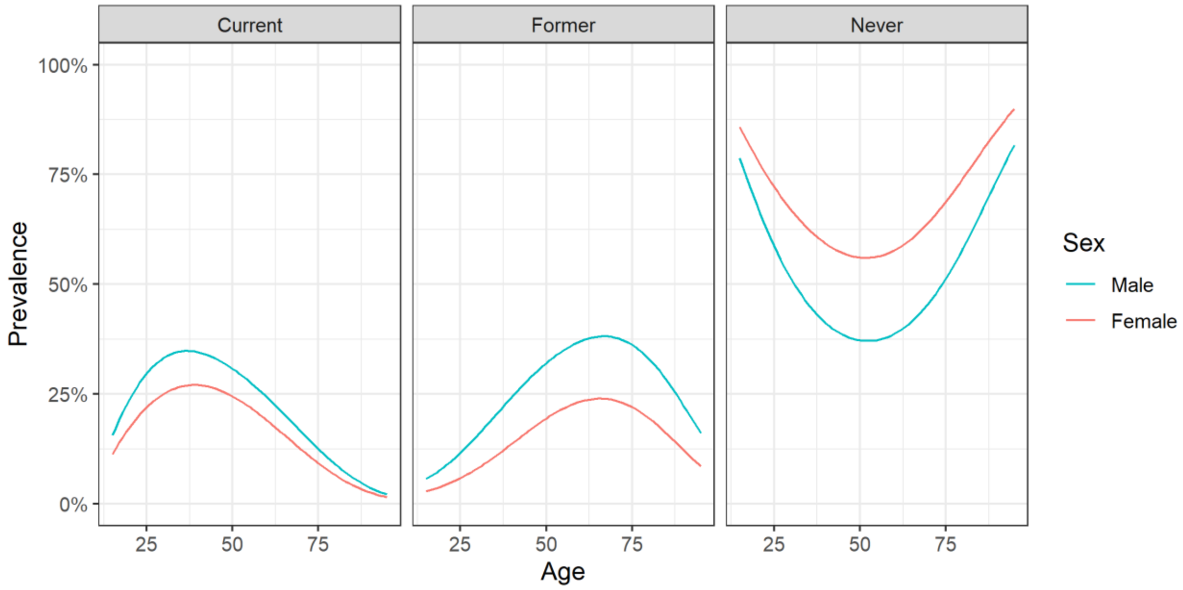

Fig. S1. Prevalence of current, former and never smokers by age and gender in Belgium.

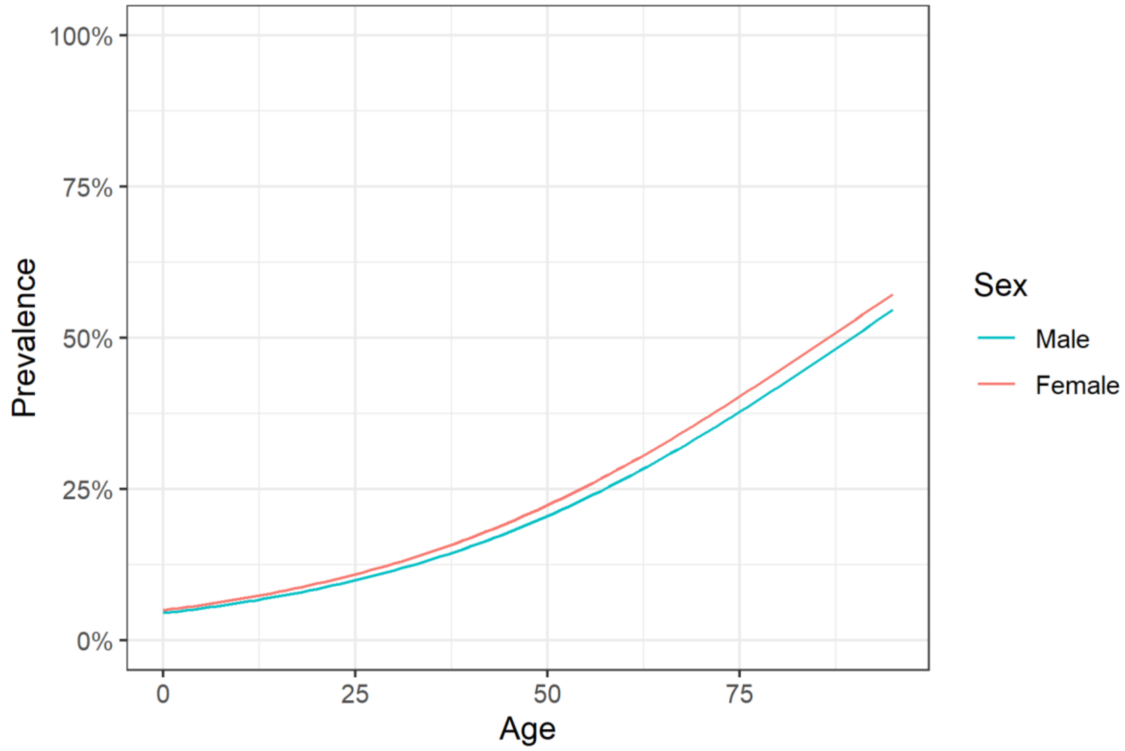

Fig. S2. Prevalence of disability based in Belgium by age and sex, based on the Global Activity Limitation Indicator (GALI).

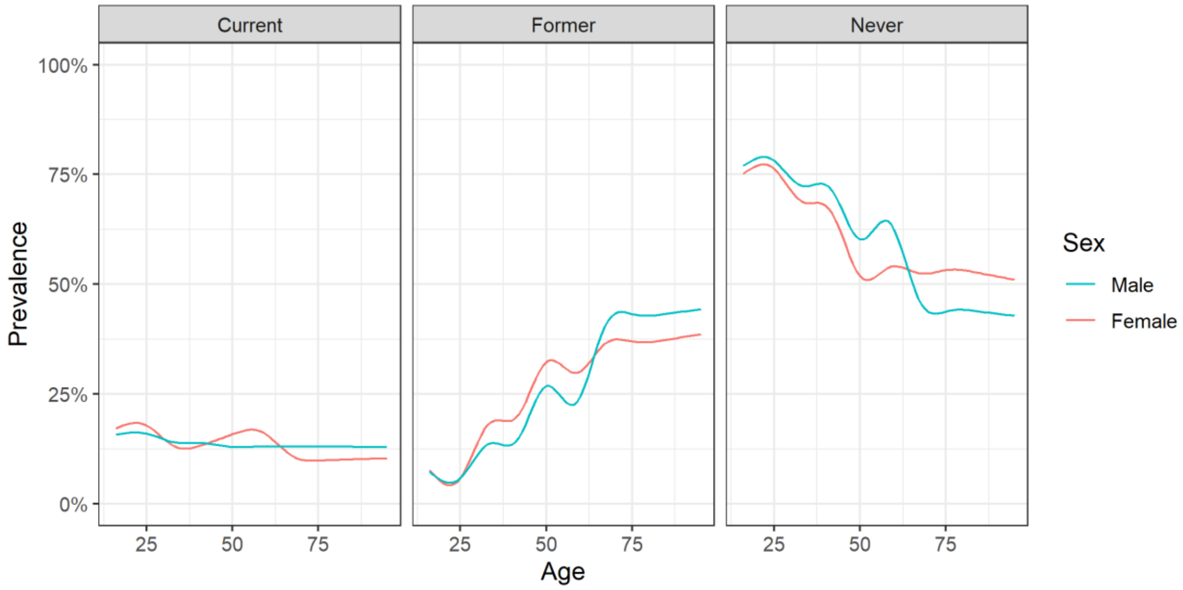

Fig. S3. Prevalence of current, former and never smokers by age and gender in Sweden.

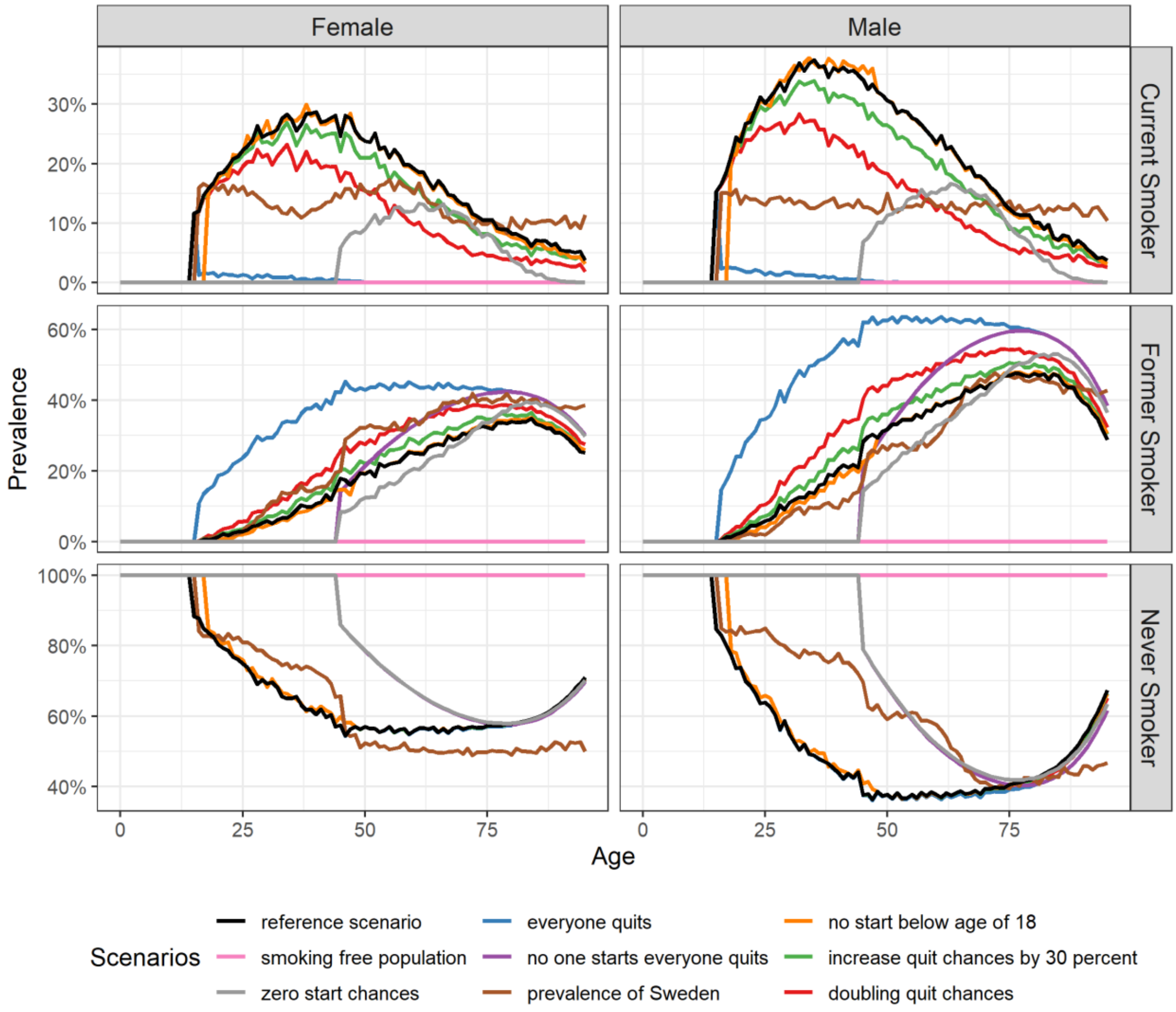

Fig. S4. Smoking prevalence by age by gender. Effect of different “what-if” scenarios and policy/intervention scenarios in Belgium, 2048.
